# Smartphone camera based assessment of adiposity: a validation study

**DOI:** 10.1038/s41746-022-00628-3

**Published:** 2022-06-29

**Authors:** Maulik D. Majmudar, Siddhartha Chandra, Kiran Yakkala, Samantha Kennedy, Amit Agrawal, Mark Sippel, Prakash Ramu, Apoorv Chaudhri, Brooke Smith, Antonio Criminisi, Steven B. Heymsfield, Fatima Cody Stanford

**Affiliations:** 1 Amazon, Inc., Seattle, WA USA; 2 Pennington Biomedical Research Center, Louisiana State University System, Baton Rouge, LA USA; 3 Departments of Medicine-Neuroendocrine Unit and Pediatrics-Endocrinology, Massachusetts General Hospital, Harvard Medical School, Boston, MA USA

**Keywords:** Obesity, Body mass index, Translational research

## Abstract

Body composition is a key component of health in both individuals and populations, and excess adiposity is associated with an increased risk of developing chronic diseases. Body mass index (BMI) and other clinical or commercially available tools for quantifying body fat (BF) such as DXA, MRI, CT, and photonic scanners (3DPS) are often inaccurate, cost prohibitive, or cumbersome to use. The aim of the current study was to evaluate the performance of a novel automated computer vision method, visual body composition (VBC), that uses two-dimensional photographs captured via a conventional smartphone camera to estimate percentage total body fat (%BF). The VBC algorithm is based on a state-of-the-art convolutional neural network (CNN). The hypothesis is that VBC yields better accuracy than other consumer-grade fat measurements devices. 134 healthy adults ranging in age (21–76 years), sex (61.2% women), race (60.4% White; 23.9% Black), and body mass index (BMI, 18.5–51.6 kg/m^2^) were evaluated at two clinical sites (*N* = 64 at MGH, *N* = 70 at PBRC). Each participant had %BF measured with VBC, three consumer and two professional bioimpedance analysis (BIA) systems. The PBRC participants also had air displacement plethysmography (ADP) measured. %BF measured by dual-energy x-ray absorptiometry (DXA) was set as the reference against which all other %BF measurements were compared. To test our scientific hypothesis we run multiple, pair-wise Wilcoxon signed rank tests where we compare each competing measurement tool (VBC, BIA, …) with respect to the same ground-truth (DXA). Relative to DXA, VBC had the lowest mean absolute error and standard deviation (2.16 ± 1.54%) compared to all of the other evaluated methods (*p* < 0.05 for all comparisons). %BF measured by VBC also had good concordance with DXA (Lin’s concordance correlation coefficient, CCC: all 0.96; women 0.93; men 0.94), whereas BMI had very poor concordance (CCC: all 0.45; women 0.40; men 0.74). Bland-Altman analysis of VBC revealed the tightest limits of agreement (LOA) and absence of significant bias relative to DXA (bias −0.42%, *R*^2^ = 0.03; *p* = 0.062; LOA −5.5% to +4.7%), whereas all other evaluated methods had significant (*p* < 0.01) bias and wider limits of agreement. Bias in Bland-Altman analyses is defined as the discordance between the y = 0 axis and the regressed line computed from the data in the plot. In this first validation study of a novel, accessible, and easy-to-use system, VBC body fat estimates were accurate and without significant bias compared to DXA as the reference; VBC performance exceeded those of all other BIA and ADP methods evaluated. The wide availability of smartphones suggests that the VBC method for evaluating %BF could play an important role in quantifying adiposity levels in a wide range of settings.

**Trial registration**: ClinicalTrials.gov Identifier: NCT04854421.

## Introduction

Body composition is associated with cardiorespiratory fitness and longitudinal health outcomes^1,2^. In clinical practice, body composition assessment is often used to evaluate dietary habits^3^, excess adiposity^4^ and malnutrition^5^, weight loss following bariatric surgery^6^, and the sarcopenia that often evolves with aging^7^. Excess adiposity impairs functional performance, is a major risk factor for developing chronic diseases, and is often accompanied by poor self-esteem^8–10^. The increased risk of chronic diseases that accompany excessive fat accumulation is the leading cause of death globally and contributes to an estimated $210 billion in medical costs in the US annually^11,12^.

In clinical practice, thresholds for body weight classifications are determined using BMI, where adults with BMI ≥25 and ≥30 kg/m^2^ are defined as overweight and obese, respectively^13–15^. However, BMI cannot discern the fat component of body mass from lean tissues. As such, adiposity levels are often misclassified in those who deviate from normalized lean mass percentages, including older adults who have lost muscle with age and athletic individuals with more muscular builds^16,17^. As studies have become more inclusive^18^, it has also become apparent that body composition, specifically percent body fat (%BF), varies across race and ethnic groups even after controlling for age and BMI, which leaves placement of weight category thresholds questionable when applied to the general public^19–22^. Due to these limitations, BMI is an imperfect obesity screening tool despite its widespread clinical application^23–26^. Alternative body composition technologies focus on measuring body fat. For example, bioelectrical impedance analysis (BIA), calipers, and anthropometric measurements are commonly used due to time and ease of measurement at the expense of accuracy^17,27–29^. Imaging techniques such as magnetic resonance (MRI), used in combination with a 4-compartment body model are considered to be the reference standard in body composition analysis due to their ability to discriminate and localize soft tissues^30,31^. Nevertheless, MRI is rarely applied in routine body composition assessment due to concerns with cost, convenience, accessibility, and equipment size. Cheaper and slightly more widely available techniques are dual x-ray absorptiometry (DXA) and computed tomography (CT). The latter two raise concerns with radiation exposure and are still too expensive and inconvenient to use routinely as a way of monitoring body composition changes at home. DXA is a popular choice as a reference method in clinical research programs^32^.

Recently, advancements in optical imaging technology have offered innovative and inexpensive methods for assessing body size, shape, and composition^33–36^. Three-dimensional imaging devices have made it possible to easily obtain thorough body measurements and estimate composition without requiring considerable skill or additional instructions^37^. However, due to their size and cost, ranging anywhere between $10,000 and $20,000 USD, current 3D optical systems remain largely unavailable to most consumers.

The gap in available, accurate and inexpensive tools for consumers to estimate and track their adiposity level led us to develop a novel imaging approach for quantifying total %BF. The application of machine learning, specifically deep learning^38^, to the task of body fat estimation from 2D optical images has not previously been explored sufficiently despite widespread potential, because of the inherent complexities in acquiring reliable reference measurements of body fat and a lack of large, annotated datasets in this domain. The study of Farina et al.^39^ is one of the few that explores the use of phone-captured digital photographs for body composition and phenotyping. However, their procedure involves photos taken in controlled environments in which participants are imaged against a uniform background. In that work, a trained operator also placed some reference markers on the image before the actual analyses began.

In contrast, when developing VBC we aimed to make the whole process completely automatic and easy enough for home use, irrespective of environment (e.g., messy kitchen, cluttered restroom, etc.) or lighting conditions.

The aim of this study was to evaluate the performance of VBC, a novel body composition analysis system, in estimating %BF directly from 2D digital photographs captured by personal smartphones, as compared to many other commercial body composition analysis methods, with DXA as the reference measurement. Note that actual DXA images are never used here; only the derived %BF measurements are used as the reference for algorithm training and for accuracy assessment. Alternative sources of reference measurements are possible, such as MRI and CT, at a greater cost.

## Results

### Participants

**Fig. 1:**
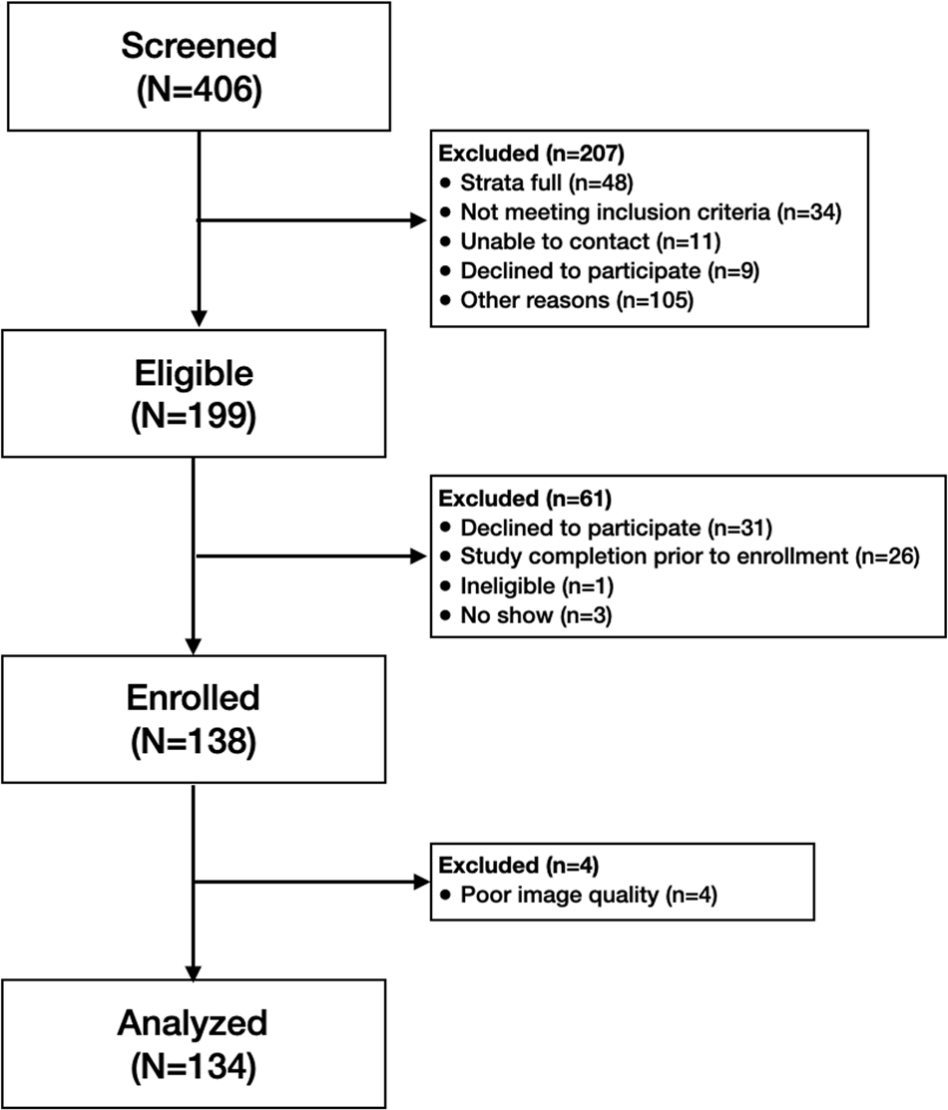
Consort diagram.

A total of 406 adults were initially screened for this study. Of those, 199 met all inclusion and exclusion criteria and were considered eligible. 138 participants were enrolled into the clinical study and 134 participants (64 from MGH and 70 from PBRC) were included in the final analysis; four participants (2.9%) were removed from the final analysis due to poor image quality (Fig. 1). The demographic and anthropometric characteristics of the final study sample are shown in Table 1. The ethnic and racially diverse sample was 60.4% White, 23.9% Black, 6.7% Asian, 3.0% Hispanic, 0.7% American Indian and the remaining 5.2% Multiracial, across the two study sites. Participant’s mean age was 43 ± 14.7 years (range, 21–76 years) and BMI 29.7 ± 6.5 kg/m^2^ (range, 18.5–51.6 kg/m^2^). DXA-measured %BF was 39.4 ± 7.2% in women and 28.6 ± 6.4% in men.

**Table 1:** Subject characteristics.

		All	Males	Females
Total number		134	52	82
	White	81 (60.4%)	31 (59.6%)	50 (61.0%)
	Black	32 (23.9%)	11 (21.2%)	21 (25.6%)
Ethnicity	Asian	9 (6.7%)	5 (9.6%)	4 (4.9%)
	Hispanic	4 (3.0%)	2 (3.8%)	2 (2.4%)
	American Indian	1 (0.7%)	0 (0%)	1 (1.2%)
	Others	7 (5.2%)	3 (5.8%)	4 (4.9%)
Age (years)		43 ± 14.7	43.1 ± 14.4	43.0 ± 14.8
Height (cm)		167.8 ± 10.2	175.6 ± 8.7	163.0 ± 7.6
Weight (kg)		84.0 ± 20.8	92.8 ± 19.7	78.3 ± 19.4
BMI (kg/m^2^)		29.7 ± 6.5	30.0 ± 5.6	29.5 ± 7.0
Waist circumference (cm)		98.5 ± 15.7	102.2 ± 14.3	96.2 ± 16.3
Hip circumference (cm)		109.4 ± 13.2	108.2 ± 11.5	110.1 ± 14.3
Waist-to-hip ratio		0.90 ± 0.08	0.94 ± 0.08	0.87 ± 0.07
DXA %BF		35.2 ± 8.7	28.6 ± 6.4	39.4 ± 7.2

*BMI* body mass index, *DXA* dual-energy x-ray absorptiometry, *%BF* percent body fat. Results are X ± SD (standard deviation).

### Body composition

VBC achieved the lowest error in estimating %BF with MAE and SD of 2.16 ± 1.54% and MAPE of 6.4% compared to DXA, with an overall bias of −0.42%. cBIA 1, 2, and 3 had bias of −0.67%, −0.12%, and −2.93%, respectively. MAE and SD for these three devices were 4.48 ± 4.01%, 4.91 ± 8.7%, and 5.85 ± 4.86%, respectively. The bias, MAE, and SD of the pBIA 1, pBIA 2, and ADP systems were −1.07%, 3.13 ± 2.10%; 0.64%, 4.72 ± 3.0%; and 0.55%, 3.14 ± 2.24, respectively. The key performance measures, including overall bias, MAE, SD, and concordance correlation coefficient (CCC) of DXA as compared to the seven devices evaluated are presented in Table 2. Compared to DXA, VBC demonstrated very high concordance (CCC = 0.96) in the overall sample, which was higher than all other methods evaluated, including ADP (*N* = 70 for ADP).

**Table 2:** Comparison of %BF estimates to DXA references stratified by sex.

	VBC	cBIA 1	cBIA 2	cBIA 3	pBIA 1	pBIA 2	ADP
All							
*N*	134	123	131	131	70	64	70
Bias (%)	−0.42	−0.67	**−0.12**	−2.93	−1.07	0.64	0.55
MAE (%)	**2.16** **±** **1.5**	4.48 ± 4.0*	4.91 ± 8.7*	5.85 ± 4.9*	3.13 ± 2.1*	4.72 ± 3.0*	3.14 ± 2.2*
MAPE (%)	**6.40**	14.20	15.40	16.70	10.30	14.00	9.70
CCC	**0.96**	0.80*	0.58*	0.72*	0.92	0.87*	0.92
Men							
*N*	52	46	50	49	27	25	27
Bias (%)	−0.17	**0.22**	1.55	−1.45	−1.41	−4.58	2.24
MAE (%)	**1.88** **±** **1.3**	4.53 ± 5.0*	6.23 ± 13.4*	4.11 ± 3.3*	3.37 ± 2.4	5.01 ± 3.0*	3.50 ± 2.4
MAPE (%)	**6.80**	18.20	22.60	15.50	13.80	17.40	12.50
CCC	**0.94**	0.57*	0.29*	0.74*	0.87	0.54*	0.88
Women							
*N*	82	77	81	82	43	39	43
Bias (%)	−0.58	−1.20	−1.15	−3.82	−0.85	3.99	**−0.51**
MAE (%)	**2.34** **±** **1.6**	4.45 ± 3.3*	4.10 ± 3.1*	6.89 ± 5.3*	2.98 ± 1.8*	4.54 ± 2.9*	2.91 ± 2.1
MAPE (%)	**6.10**	11.80	10.90	17.40	8.20	11.90	8.00
CCC	**0.93**	0.78*	0.79*	0.62*	0.91	0.79*	0.91

*ADP* air displacement plethysmography, *cBIA* consumer bio-impedance analysis, *CCC* concordance correlation coefficient, *MAE* mean absolute error, *MAPE* mean absolute percent error, *pBIA* professional bio-impedance analysis, *VBC* visual body composition. *P < 0.05 in comparison to VBC-DXA. Results are X ± s.d. Bold entries indicate best results for each row.

Further sub-cohort analyses of the performance of all devices evaluated for estimating %BF classified by sex, BMI, and ethnicity are summarized in Tables 2 and 3. When stratified by sex, VBC continues to show the lowest MAE and MAPE values. VBC has MAE ± SD 1.88 ± 1.32%, MAPE 6.8% in men and MAE ± SD 2.34 ± 1.64%, MAPE 6.13% in women. VBC also had very good concordance for both women (CCC = 0.93) and men (CCC = 0.94), as shown in Table 2.

**Table 3:** Comparison of %BF estimates to DXA references stratified by BMI and ethnicity.

	VBC	cBIA 1	cBIA 2	cBIA 3	pBIA 1	pBIA 2	ADP
BMI < 25							
*N*	34	32	33	34	23	11	23
Bias (%)	−2.23	−3.64	−3.79	−6.99	−3.34	**0.83**	−3.00
MAE (%)	**2.50** **±** **1.8**	6.36 ± 3.5	5.75 ± 3.3	7.66 ± 4.6	4.29 ± 2.2	4.41 ± 2.8	3.31 ± 2.2
MAPE (%)	**8.10**	23.00	19.60	24.50	15.70	16.80	11.90
CCC	**0.90**	0.54	0.66	0.43	0.84	0.66	0.88
BMI 25–29.9							
*N*	45	40	43	43	21	24	21
Bias (%)	**−0.30**	−1.03	0.76	−5.05	−1.90	−0.70	0.69
MAE (%)	**1.90** **±** **1.4**	3.02 ± 2.3	5.04 ± 14.3	6.00 ± 4.9	2.49 ± 1.4	3.82 ± 3.1	2.30 ± 1.9
MAPE (%)	**6.20**	10.10	17.00	17.40	8.30	12.90	7.50
CCC	**0.94**	0.86	0.23	0.39	0.92	0.84	0.92
BMI ≥ 30							
*N*	55	51	55	54	26	29	26
Bias (%)	**0.60**	1.48	1.40	1.31	1.62	1.68	3.58
MAE (%)	**2.16** **±** **1.4**	4.44 ± 4.8	4.31 ± 3.6	4.60 ± 4.6	2.63 ± 2.0	5.58 ± 2.6	3.67 ± 2.3
MAPE (%)	**5.50**	11.90	11.60	11.20	7.30	14.00	9.60
CCC	**0.95**	0.71	0.76	0.74	0.94	0.81	0.89
White							
*N*	81	73	78	78	47	34	47
Bias (%)	−0.91	−2.12	−1.09	−4.23	−1.42	**−0.30**	0.09
MAE (%)	**2.00** **±** **1.5**	4.25 ± 3.3	5.08 ± 10.7	6.00 ± 4.8	3.16 ± 2.0	3.86 ± 2.6	2.76 ± 1.9
MAPE (%)	**6.00**	13.80	16.10	17.10	10.90	11.30	9.00
CCC	**0.96**	0.83	0.47	0.69	0.92	0.90	0.93
Black							
*N*	32	31	32	32	15	17	15
Bias (%)	**0.47**	3.42	2.57	0.98	0.76	2.76	2.54
MAE (%)	**2.72** **±** **1.7**	5.06 ± 5.3	4.31 ± 3.1	5.76 ± 4.2	3.51 ± 2.0	5.31 ± 3.1	4.77 ± 2.9
MAPE (%)	**7.90**	15.90	13.30	16.00	9.60	17.20	13.30
CCC	**0.95**	0.76	0.86	0.81	0.89	0.85	0.81
All others							
*N*	21	19	21	21	8	13	8
Bias (%)	0.24	−1.22	−0.25	−3.58	−2.23	0.50	**−0.19**
MAE (%)	**1.98** **±** **1.2**	4.50 ± 4.1	5.16 ± 4.5	5.42 ± 5.8	2.23 ± 2.3	6.14 ± 3.0	2.59 ± 1.7
MAPE (%)	**5.80**	13.40	15.80	16.10	8.10	17.00	7.80
CCC	**0.96**	0.75	0.76	0.67	0.90	0.81	0.91

*ADP* air displacement plethysmography, *cBIA* consumer bio-impedance analysis, *CCC* concordance correlation coefficient, *MAE* mean absolute error, *MAPE* mean absolute percent error, *pBIA* professional bio-impedance analysis, *VBC* visual body composition. Results are X ± s.d. Bold entries indicate best results for each row.

Table 3 illustrates results stratified by BMI. Once again, in all three BMI categories VBC achieves the lowest MAE and MAPE values. BMI < 25 kg/m^2^ MAE ± SD 2.5 ± 1.8%, MAPE 8.1%. BMI 25–29.9 kg/m^2^ MAE±SD 1.9 ± 1.4%, MAPE 6.2%. BMI > 30 kg/m^2^ MAE ± SD 2.2 ± 1.4%, MAPE 5.5%. Table 3 also illustrates results stratified by race and ethnicity. VBC continues to show the lowest MAE and MAPE errors out of all methods compared in this study. White MAE ± SD 2.0 ± 1.5%, MAPE 6.0%. Black MAE ± SD 2.7 ± 1.7%, MAPE 7.9%. All others MAE ± SD 1.9 ± 1.2%, MAPE 5.8%.

**Fig. 2:**
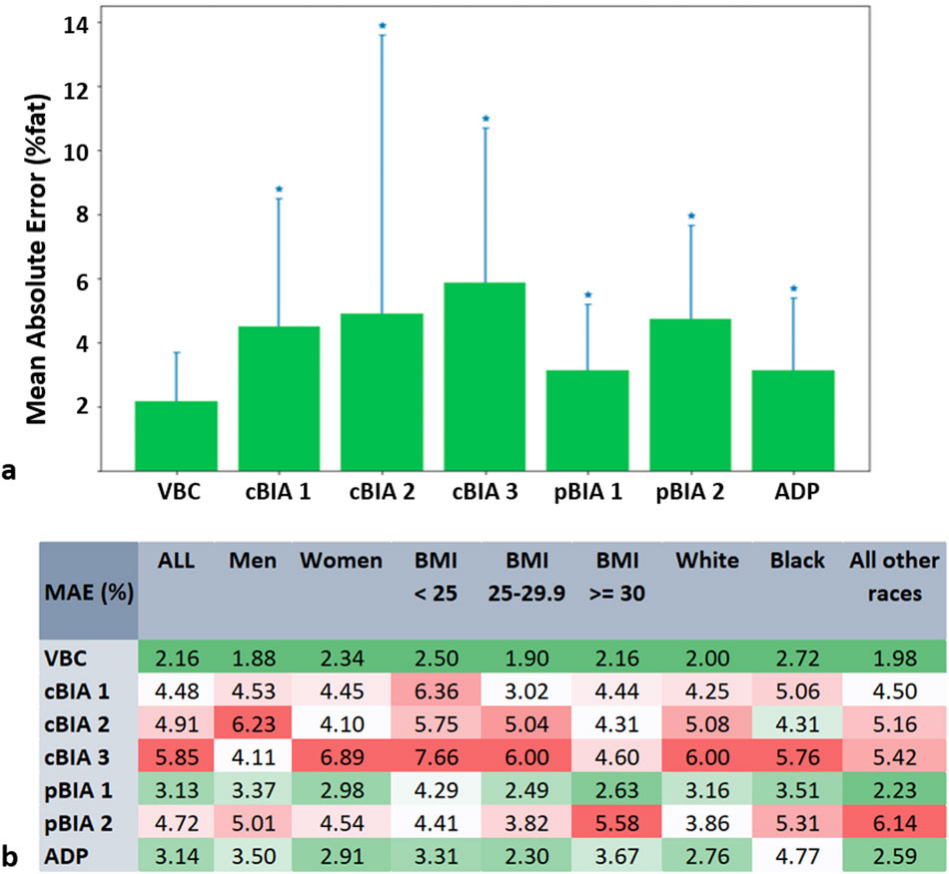
Accuracy of different measurement methods. Mean absolute errors (MAE) of the various methods evaluated with DXA as the reference **a**. MAE of various methods in comparison to DXA stratified by sex, BMI, and ethnicity **b**. The colors are interpolated linearly from green (low error) to red (high error). We defined an acceptable error range as ≤3% (dark green). This value was selected as the mean MAE for ADP and the best of the pBIA devices. Light green, white, and red shadings indicate errors outside of this range. *p < 0.05 in comparison to VBC-DXA MAE.

As shown in Fig. 2a VBC achieved the lowest overall mean absolute error in estimating %BF, which was statistically significantly better than all other methods evaluated (*p* < 0.05 for all methods), with cBIA 3 yielding the highest error. Furthermore, Fig. 2b shows a pseudo-colored representation of the mean absolute error, both overall and stratified by sex, BMI, and ethnicity (green indicates low error and red indicates high error).

**Fig. 3:**
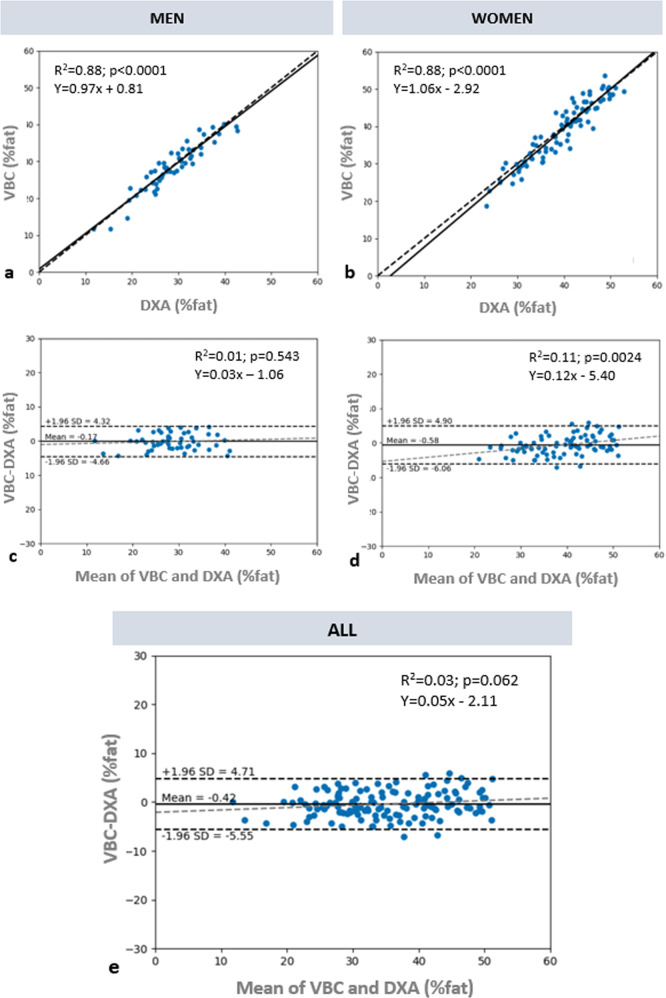
Comparison of %BF between VBC and DXA. Correlation between %BF by VBC and DXA, for **a** men and **b** women. The dashed line is identity and the solid line is the automatically fitted regression line. The two lines are very close to one another, and the correlations in both figures are significant at *p* < 0.0001. Bland-Altman analysis of the difference between %BF by VBC and DXA, for **c** men, **d** women, and **e** all. The horizontal black lines are at the mean ± 1.96 SD and the dashed gray lines are the fitted regression lines described by the equation in the panel.

**Fig. 4:**
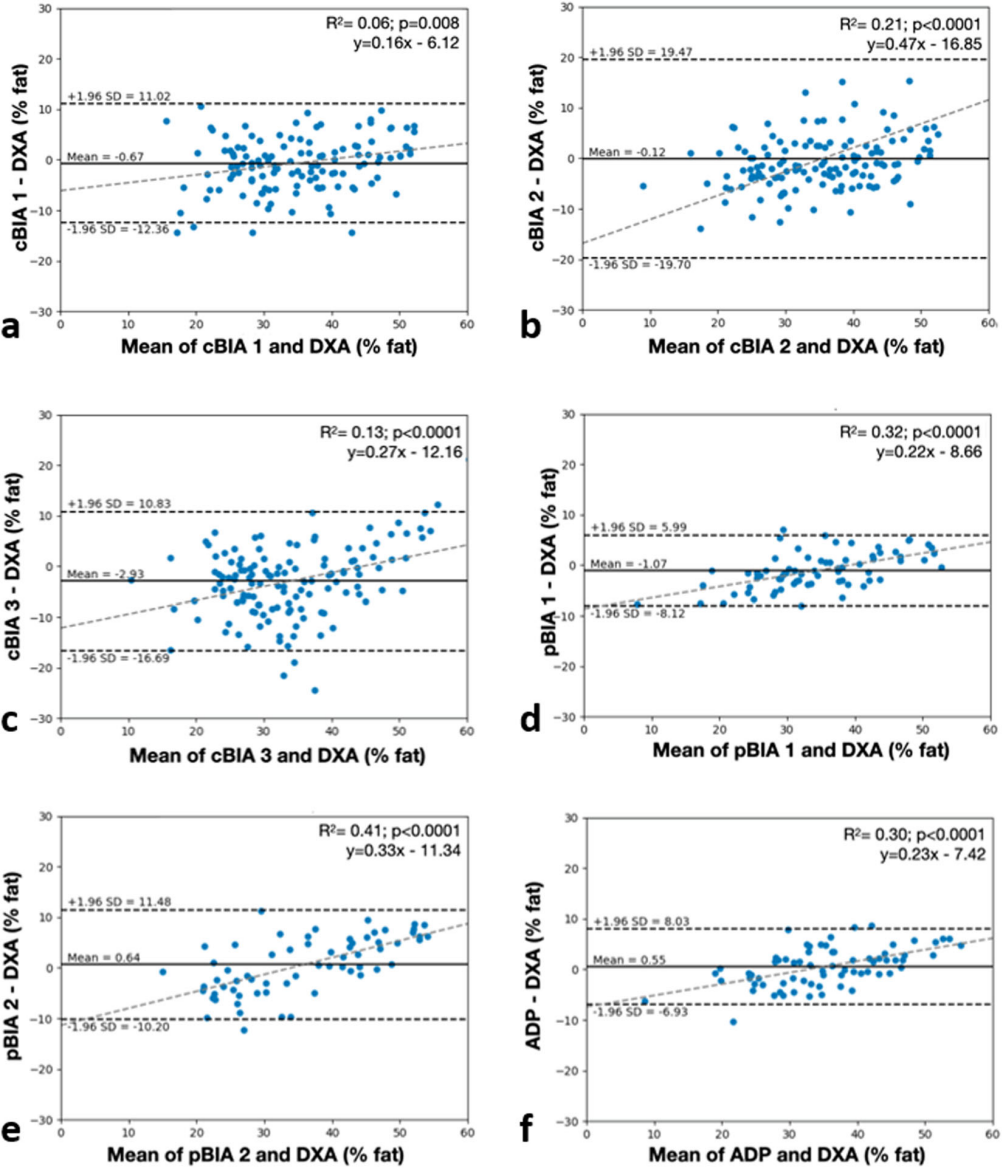
Bland-Altman analyses of the differences between %BF by DXA and the six methods evaluated for estimation of %BF. **a** cBIA 1; **b** cBIA 2; **c** cBIA 3; **d** pBIA 1; **e** pBIA 2; **f** ADP. The horizontal black lines are at the mean ± 1.96 SD and the dashed gray lines are the fitted regression lines.

Correlations between %BF evaluated by VBC and DXA among men and women are shown through scatter plots in Fig. 3a, b, respectively. VBC achieved very good correlation for both male and female participants (*R*^2^ = 0.88 for both sexes). Bland-Altman plots for VBC are also presented for men (Fig. 3c), women (3**d**) and all (3**e**). Limits of agreement for the union of men and women are −5.55%, +4.71%. The Bland-Altman plot for men shows nearly no bias, while that for women shows a small bias (~1% at the extremes), which is not really clinically or personally significant. By contrast, larger levels of bias are present in all other methods (Fig. 4). Individual level validity for all other methods is presented using Bland–Altman plots in Fig. 4a–f. VBC achieves the tightest limits of agreement without any statistically significant bias, whereas all other methods had significant bias (*p* < 0.05) and wider limits of agreement.

**Fig. 5:**
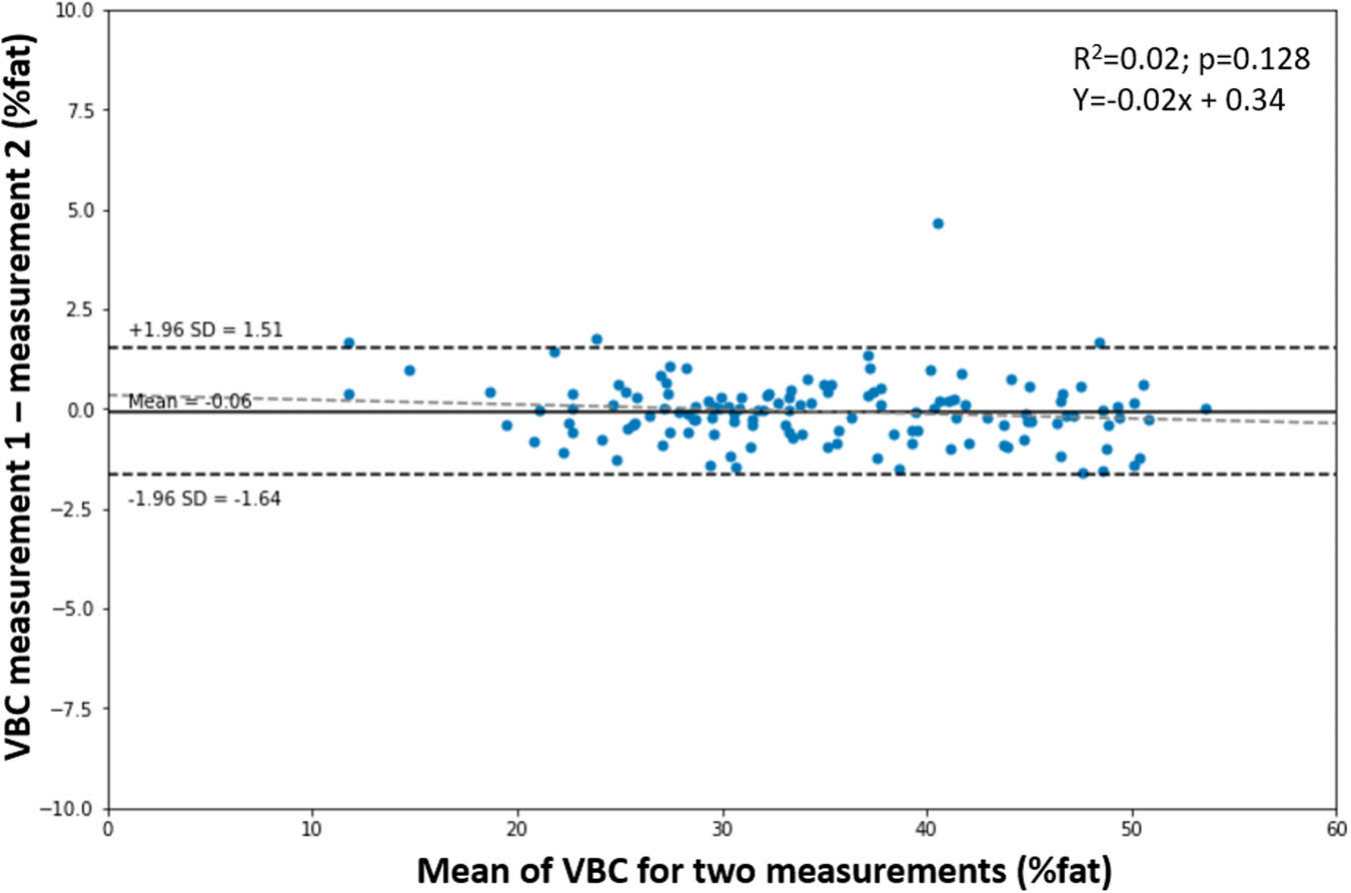
Bland-Altman analysis of repeated VBC measurements to assess technical error.

Finally, Fig. 5 shows repeatability of VBC measurements (a.k.a. technical error). For each participant we have double measurements for: VBC, cBIA1, cBIA2 and cBIA3. The Bland-Altman plot shows the mean of two VBC measurements in the X axis and their difference in Y. Good VBC repeatability is indicated by very tight Limits of Agreement (−1.64%, +1.51%) and high *R*^2^ (0.99). For comparison, the Limits of Agreement for the cBIA devices are, respectively: (−3.56%, +3.74%), (−31.06%, +33.84%) and (−0.20%, +0.23%) and their *R*^2^ values are: 0.97, 0.27, 1.00.

## Discussion

There is a need for an accurate, easy-to-use, and widely accessible tool for assessment of body composition outside of specialized research facilities. The current study evaluated the performance of a novel computer-vision based algorithm for estimating %BF from 2D smartphone photographs. Our findings support the validity of VBC in estimating %BF relative to DXA, the reference used in this study. VBC had the lowest MAE (2.16 ± 1.54%), highest overall concordance with DXA (Men CCC, 0.94; Women CCC, 0.93), and the tightest limits of agreement (LOA, −5.5%, +4.7%) among the evaluated devices including several BIA systems and ADP.

While multiple other devices are available for capturing a person’s image and transforming the quantified information into an estimate of body composition^40,41^ VBC needs only two conventional photographs of the participant captured via their personal smartphone camera. These two images are securely sent to the cloud where they are: (1) segmented into person and background, and (2) passed onto a CNN model that automatically analyzes the images, extracts visual features relevant to body composition and generates an estimate of %BF. Carletti et al. described a similar framework to directly estimate %BF from depth images^42^. In contrast, VBC does not require specialized or expensive equipment like depth cameras, but instead, works with conventional smartphone cameras, making it an accessible tool at the consumer level.

Camera-enabled smartphones are widely popular, with over 2.5 billion users worldwide^43^. As such, there is a potential for a tool like VBC to have wide scale use to better quantify and monitor BF% in persons across weight classes. Given its ease of use and low-cost, people can readily measure their body fat; for instance, biweekly or monthly, and correlate its temporal trend with their lifestyle habits, such as physical activity, dietary changes and sleep patterns.

VBC outperformed commercial single frequency BIA systems for home use as they only capture the leg–leg electrical pathway and are known to have limited accuracy due to several factors that include variable participant hydration and use of population-specific %BF prediction equations^44,45^. The evaluated multi-frequency whole-body pBIA systems overcome some of the limitations present in the cBIA devices, although VBC still outperformed them both. The Bod Pod ADP device evaluated at the PBRC site is a recognized reference method for some types of studies, notably those in which radiation exposure is a concern, and at centers without available DXA systems^46^. As with the other evaluated devices, VBC also outperformed Bod Pod when using DXA as the reference in the current study. Some of the differences between VBC, BIA and ADP have to do with the underlying models (2-compartment vs 4-compartment) and the (often proprietary) prediction equations. Note that VBC does not use a single, hand-designed equation, but a complex, highly non-linear, automatically optimized function expressed in CNN form. Given these initial findings, the VBC method appears to function at least on par, if not better, than professional systems such as pBIA and ADP.

While VBC performed well in the current study, several limitations of the device and our study should be noted. The CNN model was trained with photos of people wearing minimal, form-fitting clothing. Wearing full length sleeves, pants, shorts covering parts of the stomach, abdomen or thighs, or loose clothing may yield inaccurate results. Extremely dark or bright images can hide important visual information and reduce the model accuracy. Other variables that may cause inaccuracies are extreme camera tilt, camera positioned too far from the participant, holding the belly in, scanning after a large meal or an intense workout, flexing muscles or large deviations from the canonical “A” pose. The VBC model does not generate %BF estimates above 64%. The model produces a single number for %BF estimation, but currently does not provide any details on fat localization. For instance, it does not differentiate between visceral and subcutaneous adipose tissue.

As a reference we use DXA because of its popularity in similar clinical studies and its reasonable accuracy. We acknowledge the potential systematic bias of using DXA on which the algorithm or CNN was trained to assign labels to VBC images, on the results of agreement with DXA in the test set. An MR-based 4-model reference may have produced a more accurate reference at an increased cost and reduced sample size.

The study was limited to 134 participants with weight less than 400 lbs (181 kg); a larger and more diverse sample may have further strengthened our findings. However, the study did have enough power to reach statistical significance for the primary outcome of evaluating the performance of VBC and various other methods against DXA as the reference standard.

This study presents the first validation of a novel, accessible, and easy-to-use system for estimating an individual’s total body fat using only two photographs taken with a conventional smartphone. The VBC method had the lowest mean absolute error and standard deviation and the tightest limits of agreement when compared to six commercially available tools. Percent fat estimated by VBC also had stronger concordance with those by DXA compared to the other methods and BMI. No significant bias was present for VBC relative to DXA according to a Bland–Altman analysis. These results support the use and feasibility of VBC for at-home measurement and monitoring of total body fat.

## Methods

### Trial design and oversight

**Fig. 6:**
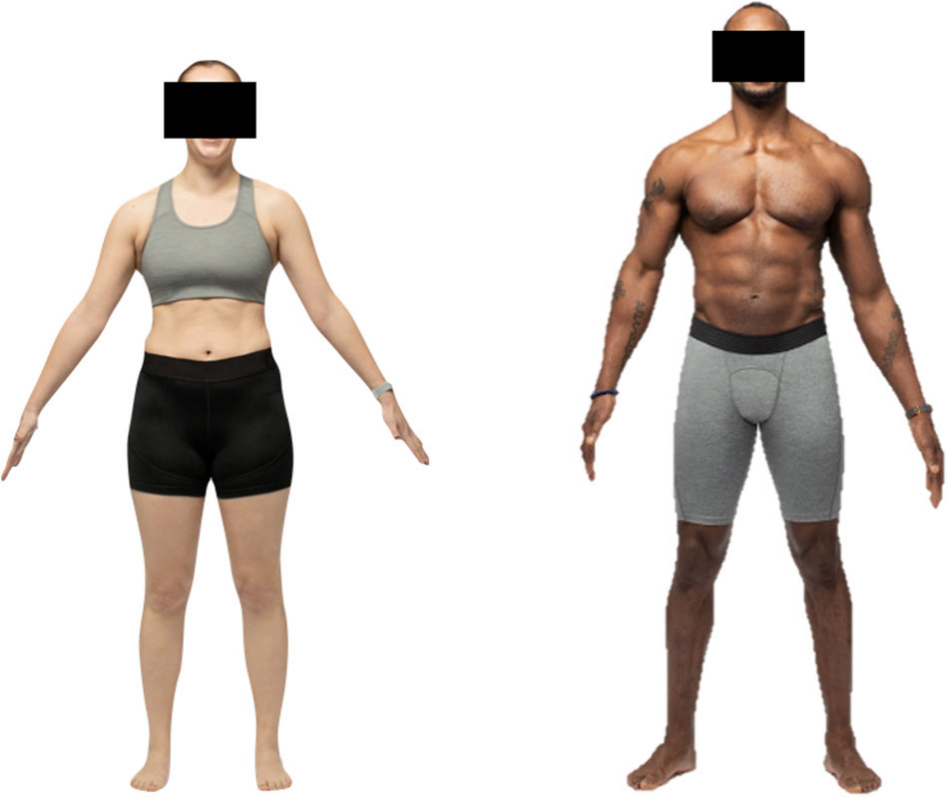
Example smartphone images. The VBC algorithm requires images of the user’s front and back while wearing minimal clothing and holding an “A” pose as inputs to the model.

The VBC analysis system was examined in a prospective, clinical validation study conducted at two clinical trial sites: Massachusetts General Hospital (MGH), Harvard University, and Pennington Biomedical Research Center (PBRC), Louisiana State University. The study protocol was approved by the Advarra Institutional Review Board (Columbia, MD) as well as the MGH and PBRC Institutional Review Boards. All participants provided written informed consent. The authors also affirm that human research participants provided informed consent for publication of the images in Fig. 6.

Participants were contacted by a recruiter who performed pre-screening based on demographic information as well as inclusion and exclusion criteria. Eligible participants were asked to arrive at their respective facility for a single 2–3-h visit following a 4-h fast. Upon arrival, they were provided a copy of the consent form and a private room for the consenting process. Those who agreed to participate completed the following assessments for %BF: DXA and VBC scans, three consumer-grade bio-impedance analysis (cBIA) smart scale evaluations, two professional BIA (pBIA) system evaluations, and air displacement plethysmography (ADP), in that order. ADP was performed only at PBRC. Women with reproductive potential also completed a urine pregnancy test prior to undergoing these assessments.

### Trial participants

Participants were healthy adults recruited using a combination of web-based questionnaires, direct phone calls, media, and advertising in local communities. Included men and women were generally in good health, between the ages of 21 and 80 years, weighed less than 400 lbs (181 kg), and willing to comply with study procedures. Potential participants were excluded if they had medical implants such as a pacemaker or a total knee replacement or had previously undergone body altering procedures such as arm or leg prosthesis, amputation, or breast augmentation. Participants were also excluded if they took loop diuretics within 6 h of their scheduled visit, had a diagnosis of heart failure, or were undergoing active cancer treatment, see Fig. 1 for our consort diagram.

### Trial procedures

For each of the participants, trained facility staff acquired the following data: demographic information such as age, sex, ethnicity, height, and weight; circumference measurements taken at the waist, hip, arm, and thigh; 2D photographs captured by a smartphone camera; %BF estimates from consumer and professional BIA scales, ADP, and DXA; only participants at PBRC underwent ADP (*N* = 70). Note that DXA images were never used, only their derived %BF.

### Anthropometry

Circumference measurements were taken at the waist, hip, arm, and thigh by trained staff at conventional anatomic locations. Measurements were recorded in centimeters. Body circumferences were acquired to ensure a good distribution of body sizes and shapes. However, those measurements were not used in the VBC algorithm.

### VBC scan

Participants were dressed in minimal, form-fitting clothing (Fig. 6) without socks, shoes, or any protruding wearables (watches, jewelry, etc.), such that the mid-thigh and belly button areas were visible to the smartphone camera. Each participant was asked to stand in an “A” pose and then had four photographs (front, back, left-side, and right-side profiles) taken with an iPhone-10 (Apple, Inc.) front-facing camera with their faces out of frame.

### Computer vision model

**Fig. 7:**
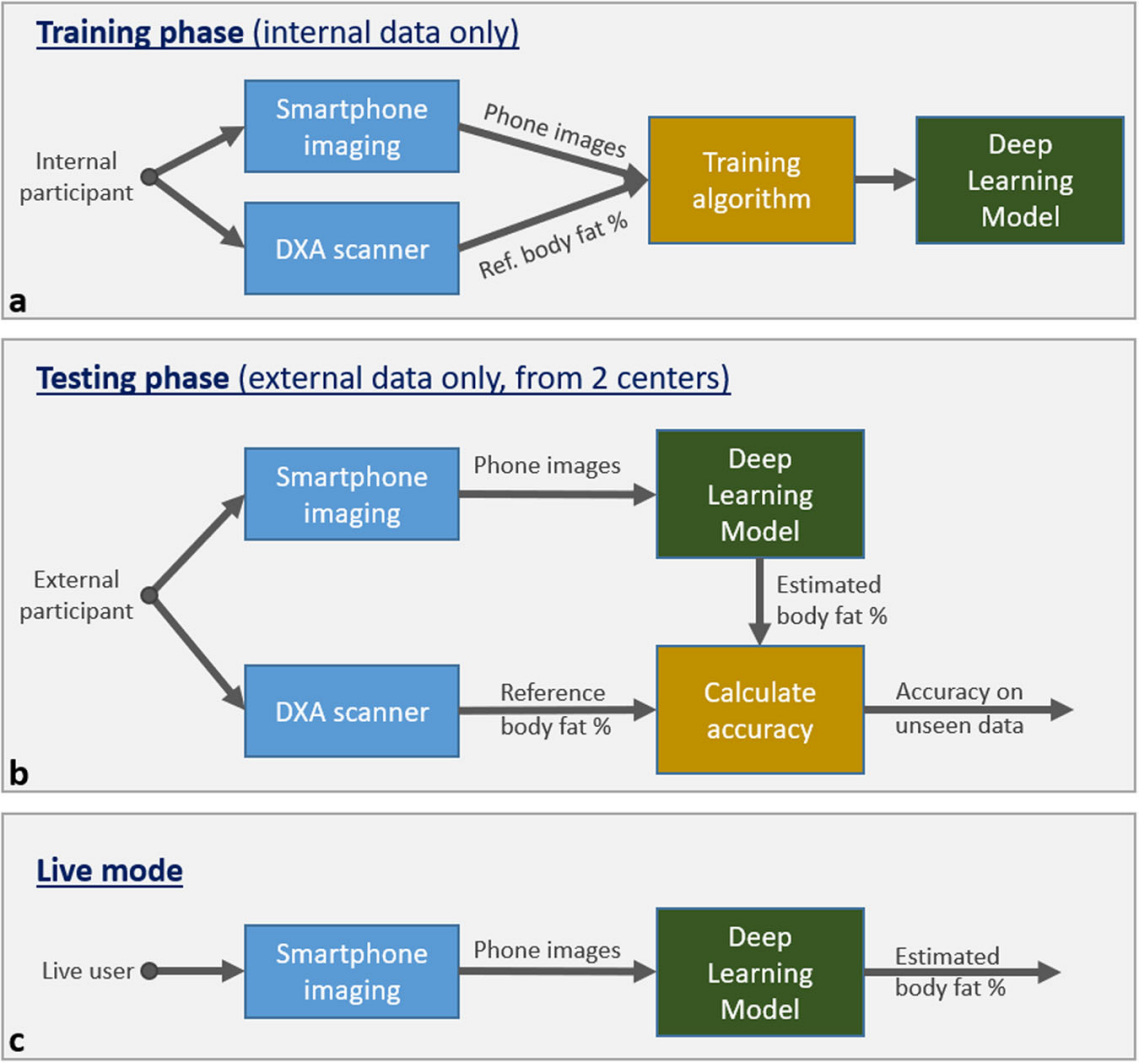
Different phases of our algorithm. Block diagrams illustrating the data flow for different phases of our algorithm: training **a**, testing **b**, and live use **c**. DXA images are never used.

The body composition estimation algorithm consists of a bespoke convolutional neural network (CNN)^47,48^ that was optimized (trained) on internal data (not the external trial data) to estimate %BF directly from two input photographs (front and back) of the user standing in an A pose as shown in Fig. 6. The algorithm does not need 3D scans nor professional-quality photographs. Photos taken with personal smartphones suffice. Photos were acquired through a smartphone positioned 4–6 feet away from the participant above their knee height. The algorithm was trained to be robust to occlusions (e.g., from furniture), cropping (e.g., feet or head out of frame) and varying participant-phone distances. For the training set, both iPhones and Android phones are used, to ensure generalization. Of note, the side images are used to generate a three-dimensional body model (using a different computer vision algorithm), which is a feature of the commercial product (Amazon Halo); but those images are not used for estimation of %BF. The VBC %BF algorithm was developed using the Python programming language (Python Software Foundation; available at www.python.org) and uses the PyTorch machine learning framework (available at www.pytorch.org and maintained by Meta) for training and evaluating the CNN. The developed model was trained on machines with modern graphic processing units (GPU) for speed. Figure 7 illustrates the training and the accuracy assessment (validation) phases.

### Annotated datasets

We have collected two datasets: an interna; training dataset and an external testing dataset. The training dataset has multiple participants covering a wide range of BMI, body fat, body shapes, camera angles, camera distance, illumination conditions, environments etc. The testing dataset is external, collected at the two participating trial sites. Each data entry consists of: (1) Four smartphone images of the participant in an “A” pose, and (2) Associated reference measurements for total body fat percentage. The two datasets are completely disjoint and no test data was ever used during training or algorithm optimization.

#### Person-Background segmentation

The first step in the processing chain is to separate the foreground person from the background. This is achieved by a bespoke CNN that was trained on a pixel-wise labelled subset of the training set to achieve >98% test pixel-wise accuracy. After background removal, the front and back photos are normalized to a canonical size to remove the effect of varying camera distance (perspective foreshortening).

#### Training to estimate body fat

Using the normalized front and back photos as inputs for training, a second CNN model was pre-trained to automatically extract discriminative visual features (shape, texture) relevant to body composition. Notice that with modern deep learning techniques it is no longer necessary to design visual features (filter banks etc.) by hand. The architecture of our CNN uses multiple convolutional blocks with additional branches for multi-scale feature extraction. The multi-scale extension allows the network to automatically select and utilize high resolution image features and capture fine details (e.g., skin bumps) across the body fat spectrum. This CNN is trained to be resilient to noise in the input images, robust to expected variations in illumination and camera orientation, and to work across different phone models, camera devices and color-spaces (grayscale/RGB). Next, transfer learning is applied to fine-tune the initial, pre-trained model using DXA %BF training data. DXA here is used only to provide reference measurements of body fat, and DXA images are never used.

#### Accuracy validation

In the accuracy assessment stage (Fig. 7), for each trial participant a pair of front and back photos are sent into the trained Deep Learning Model and the output is the estimated %BF measurement. The accuracy of these estimates is calculated on the test set, by comparison with DXA-obtained reference %BF measurements.

#### Runtime (live mode)

During live use, only the top branch of the validation flow diagram (Fig. 7) is used as there are no reference measurements.

### Why use DXA measurements as reference?

DXA measures fat mass, i.e. triglyceride, while conventional MRI measures adipose tissue volume. Most body fat is stored in adipose tissue, but not all, thus MRI and DXA do not measure exactly the same thing^49^. Fat mass is what most physiologists are interested in as it connects with energy balance. Both the MRI and the DXA fat estimation paths have errors: (1) in MRI because tissue volumes need to be segmented in 3D, most often by hand, (2) in DXA because 3D mass information is extrapolated from 2D x-ray images. Also, capturing full-body MR images at a useful resolution takes many minutes. Finally, DXA represents a good quality-cost-speed compromise, as demonstrated by its popularity in clinical research^50^. Since no radiological images are used anywhere in our algorithms, extracting reference body fat measurements from multiple sources is an alternative.

### Why use front and back images?

In our approach there is no manual design and selection of visual features. However, we have observed empirically that the algorithm yields the highest accuracy when the front and rear images are used, and the side views do not have much influence on the final accuracy.

### Dual-Energy X-ray absorptiometry

Total body fat was measured on each participant in the test set with a Hologic Discovery A or Hologic Horizon A DXA system (Hologic, Inc., Marlborough, MA, USA). Both DXA systems were calibrated and operated according to manufacturer guidelines. Attired in minimal clothing, participants were asked to lay flat on the DXA table for about 10 min while the device performed the scan. The participants are ensured to fit entirely within the DXA field of view. All scans were evaluated with Hologic Apex software version 5.6 and the National Health and Nutrition Examination Survey (NHANES) Body Composition Analysis calibration feature was disabled.

### Bioimpedance analysis

Three consumer weight scales capable of BIA-based body composition analysis were included in the protocol: FitBit Aria 2 (Fitbit, San Francisco, CA); Tanita BF-684W (Tanita, Tokyo, Japan); and Renpho ES-24M-W/B (Joicom Corporation, Anaheim, CA). These scales are designated as cBIA 1, cBIA 2, and cBIA 3, respectively, in the sections that follow. These scale models were chosen based on their popularity with customers. We decided to use multiple BIA scales to assess the level of discordance between their measurements for the same participant. All scales were available at both trial sites. Participants were weighed in duplicate on the consumer scales, and the results were averaged for analyses. All participants also underwent professional BIA (pBIA) at PBRC with an InBody S10 (InBody Co., Seoul, Korea) and at MGH with a RJL system (Quantum IV, RJL Systems, Clinton Township, MI, USA.). The RJL system uses a single frequency (50 Khz) and four gel adhesive electrodes. Instead, InBody is multi-frequency and uses contact electrodes. InBody and RJL are designated as pBIA1 and pBIA 2, respectively, in the sections that follow and were analyzed separately. Both InBody S10 and RJL Quantum IV use a tetrapolar 8-point tactile electrode system. The device measures impedance, resistance and reactance in body segments at multiple frequencies. Each participant was measured once following cleaning of the electrodes with alcohol.

### Air displacement plethysmography

Participants who were evaluated at PBRC (*N* = 70) also had %BF assessed with the BOD POD ADP device (BodPod Gold Standard Body Composition Tracking System, COSMED, Rome, Italy). In addition to the specific form-fitting clothing for this study, participants put on a swim cap before entering the device. The BOD POD body composition test was performed once with each evaluation including two measurements of body volume that were averaged and then corrected for thoracic gas volume using the system software (v4.5.0). Fat mass and %BF were calculated from body density by BOD POD software using Siri’s equation^51^. We include ADP and BIA body fat estimations here for comparison, however, it should be clear that those estimates depend strongly on the exact nature of the prediction equations used within the device.

### Statistical methods

Descriptive statistics were computed for the participant characteristics stratified by sex, where appropriate. Fixed bias (or mean error) was calculated as the difference between %BF_DXA_ and %BF estimates from all other methods evaluated: VBC, cBIA1–3, pBIA1–2, and ADP. Mean absolute error (MAE), standard deviation (SD) of absolute error, and mean absolute percent error (MAPE) were calculated for all %BF estimates and stratified by sex, BMI, and race. Wilcoxon signed rank test was used to compare matched samples to assess whether their population mean ranks differ (i.e., paired difference test) for the overall study population and stratified by sex. Pearson correlation and Lin’s concordance correlation coefficient (CCC) between DXA and all other methods were also calculated and stratified by sex. The method of Meng et al.^52^ was used to determine whether VBC was significantly better correlated to the criterion method of DXA compared to the cBIA1–3, pBIA1–2, and ADP measurements. Bland-Altman plots were used to determine the mean difference and 95% limits of agreement (LOA) between DXA reference standard and VBC as well as all other methods. No adjustment for multiple comparisons. All analyses were conducted using Microsoft Excel (Microsoft, Inc., Redmond, WA) and Python. Significance was set at an alpha level of 0.05, 2-tailed.

### Reporting summary

Further information on research design is available in the Nature Research Reporting Summary linked to this article.

## Supplementary information

Reporting Summary

## Data Availability

The third-party data use agreements from our multi-site clinical study does not allow for its distribution to the public. Furthermore, these data are proprietary and will be used for commercialization of research findings. However, the authors can provide access to the final results upon request for verification of our findings. Furthermore, our results can be verified and extended by capturing a dataset of at least 100 participants as per the capture protocol described in the manuscript.

## Code availability

Code not available for public access. All data analysis and visualizations were done in Python and Microsoft Excel.

## References

[1] Després JP (2012). Body fat distribution and risk of cardiovascular disease: an update. Circulation.

[2] Church TS, LaMonte MJ, Barlow CE, Blair SN (2005). Cardiorespiratory fitness and body mass index as predictors of cardiovascular disease mortality among men with diabetes. Arch. Intern. Med..

[3] Howe AS (2013). Dieting status influences associations between dietary patterns and body composition in adolescents: a cross-sectional study. Nutr. J..

[4] Gallagher D (2000). Healthy percentage body fat ranges: an approach for developing guidelines based on body mass index. Am. J. Clin. Nutr..

[5] Wells JCK (2019). Body composition of children with moderate and severe undernutrition and after treatment: a narrative review. BMC. Med..

[6] de Aquino LA, Pereira SE, de Souza Silva J, Sobrinho CJ, Ramalho A (2012). Bariatric surgery: impact on body composition after Roux-en-Y gastric bypass. Obes. Surg..

[7] Evans WJ, Campbell WW (1993). Sarcopenia and age-related changes in body composition and functional capacity. J. Nutr..

[8] Hales C. M., Carroll M. D., Fryar C. D. & Ogden C. L. Prevalence of Obesity and Severe Obesity Among Adults: United States, 2017–2018. *NCHS Data Brief*. 1–8 (2020). 32487284

[9] Must A (1999). The Disease Burden Associated With Overweight and Obesity. JAMA.

[10] Papadopoulos, S. & Brennan, L. Correlates of weight stigma in adults with overweight and obesity: A systematic literature review. *Obesity*. 10.1002/oby.21187. (2015). 10.1002/oby.21187 26260279

[11] Finkelstein EA, Trogdon JG, Cohen JW, Dietz W (2009). Annual medical spending attributable to obesity: payer-and service-specific estimates. Health Aff. (Millwood).

[12] Wang YC, McPherson K, Marsh T, Gortmaker SL, Brown M (2011). Health and economic burden of the projected obesity trends in the USA and the UK. Lancet..

[13] (CDC). Defining Adult Overweight and Obesity. At https://www.cdc.gov/obesity/basics/adult-defining.html. (2020).

[14] Jensen MD (2014). 2013 AHA/ACC/TOS guideline for the management of overweight and obesity in adults: a report of the American College of Cardiology/American Heart Association task force on practice guidelines and the obesity society. Circulation.

[15] Wharton S (2020). Obesity in adults: a clinical practice guideline. CMAJ.

[16] Wong JC, O’Neill S, Beck BR, Forwood MR, Khoo SK (2021). Comparison of obesity and metabolic syndrome prevalence using fat mass index, body mass index and percentage body fat. PLoS One.

[17] Sommer I (2020). The performance of anthropometric tools to determine obesity: a systematic review and meta-analysis. Sci. Rep..

[18] Goacher PJ, Lambert R, Moffatt PG (2012). Can weight-related health risk be more accurately assessed by BMI, or by gender specific calculations of Percentage Body Fatness?. Med. Hypotheses.

[19] Heymsfield SB, Peterson CM, Thomas DM, Heo M, Schuna JM (2016). Why are there race/ethnic differences in adult body mass index-adiposity relationships? A quantitative critical review. Obes. Rev..

[20] Stanford FC, Lee M, Race HC (2019). Ethnicity, sex, and obesity: is it time to personalize the scale?. Mayo Clin. Proc..

[21] Wildman RP, Gu D, Reynolds K, Duan X, He J (2004). Appropriate body mass index and waist circumference cutoffs for categorization of overweight and central adiposity among Chinese adults. Am. J. Clin. Nutr..

[22] Byrd AS, Toth AT, Stanford FC (2018). Racial Disparities in Obesity Treatment. Curr. Obes. Rep..

[23] Sommer I (2020). The performance of anthropometric tools to determine obesity: a systematic review and meta‑analysis. Sci. Rep..

[24] Nuttall FQ (2015). Body mass index: obesity, BMI, and health: a critical review. Nutr. Today.

[25] Kok P, Seidell JC, Meinders AE (2004). The value and limitations of the body mass index (BMI) in the assessment of the health risks of overweight and obesity. Ned. Tijdschr. Geneeskd..

[26] Freedman DS, Sherry B (2009). The validity of BMI as an indicator of body fatness and risk among children. Pediatrics.

[27] Lee SY, Gallagher D (2008). Assessment methods in human body composition. Curr. Opin. Clin. Nutr. Metab. Care.

[28] Borga M (2018). Advanced body composition assessment: from body mass index to body composition profiling. J. Investigative Med..

[29] Klein S (2007). Waist circumference and cardiometabolic risk: a consensus statement from Shaping America's Health: Association for Weight Management and Obesity Prevention; NAASO, The Obesity Society; the American Society for Nutrition; and the American Diabetes Association. Am. J. Clin. Nutr..

[30] Prado CM, Heymsfield SB (2014). Lean tissue imaging: a new era for nutritional assessment and intervention. JPEN J. Parenter. Enter. Nutr..

[31] Smith S, Madden AM (2016). Body composition and functional assessment of nutritional status in adults: a narrative review of imaging, impedance, strength and functional techniques. J. Hum. Nutr. Diet..

[32] Sheperd J, Ng B, Heymsfield SB (2017). Body Composition by DXA. Bone.

[33] Harty PS (2020). Novel body fat estimation using machine learning and 3-dimensional optical imaging. Eur. J. Clin. Nutr..

[34] Tinsley GM, Moore ML, Dellinger JR, Adamson BT, Benavides ML (2020). Digital anthropometry via three-dimensional optical scanning: evaluation of four commercially available systems. Eur. J. Clin. Nutr..

[35] Heymsfield SB (2018). Digital anthropometry: a critical review. Eur. J. Clin. Nutr..

[36] Sobhiyeh S (2020). Digital anthropometry for body circumference measurements: Toward the development of universal three-dimensional optical system analysis software. Obes. Sci. Pr..

[37] Cabre, H. E. et al. Validity of a three-dimensional body scanner: comparison against a 4-compartment model and dual energy X-ray absorptiometry. *App. Phys. Nutr. Metab*. Accepted Manuscript. 10.1139/apnm-2020-0744 33320733

[38] LeCun Y, Bengio Y, Hinton G (2015). Deep learning. Nature.

[39] Farina G (2016). A Smartphone application for personal assessments of body composition and phenotyping. Sens. (Basel).

[40] Adler C (2017). Validity and reliability of total body volume and relative body fat mass from a 3-dimensional photonic body surface scanner. PLoS ONE.

[41] Tinsley GM, Moore ML, Benavides ML, Dellinger JR, Adamson BT (2020). 3-Dimensional optical scanning for body composition assessment: A 4-component model comparison of four commercially available scanners. Clin. Nutr..

[42] Carletti, M. et al. Analyzing Body Fat from Depth Images. *2018 International Conference on 3D Vision (3DV)*, Verona, 2018, pp. 418–425,

[43] Shoukat S (2019). Cell phone addiction and psychological and physiological health in adolescents. EXCLI J..

[44] Leahy S, O’Neill C, Sohun R, Jakeman P (2012). A comparison of dual energy X-ray absorptiometry and bioelectrical impedance analysis to measure total and segmental body composition in healthy young adults. Eur. J. Appl Physiol..

[45] Pateyjohns IR, Brinkworth GD, Buckley JD, Noakes M, Clifton PM (2006). Comparison of three bioelectrical impedance methods with DXA in overweight and obese men. Obes. (Silver Spring).

[46] Lowry DW, Tomiyama AJ (2015). Air displacement plethysmography versus dual-energy x-ray absorptiometry in underweight, normal-weight, and overweight/obese individuals. PLoS One.

[47] Krizhevsky A, Sutskever I, Hinton G (2012). ImageNet classification with deep convolutional neural network. Adv. Neural Inf. Process. Syst..

[48] Dabiri S (2020). Deep learning method for localization and segmentation of abdominal CT. Comput Med Imaging Graph.

[49] Hübers M (2019). Association between fat mass, adipose tissue, fat fraction per adipose tissue, and metabolic risks: a cross-sectional study in normal, overweight, and obese adults. Eur. J. Clin. Nutr..

[50] Kullberg J (2009). Whole-body adipose tissue analysis: comparison of MRI, CT and dual energy X-ray absorptiometry. Br. J. Radio..

[51] Siri, W. E. 1961 Body composition from fluid spaces and density: analysis of methods. In: Brozek J., Henschel A. (eds) Techniques for Measuring Body Composition. National Academy of Sciences/National Research Council, Washington, DC, pp. 223–224.

[52] Rosenthal R, Rubin D, Meng X-L (1992). Comparing correlated correlation coefficients. Psychological Bull..

